# Montana

**Published:** 1897-03

**Authors:** 


### A Bill for An Act to Provide for the Creation of a Veterinary Examining Board for the Protection of the Live-stock Interests of the State of Montana.

*Be it Enacted by the Legislative Assembly of the State of Montana:*

That the Governor, with the advice and consent of the Senate, shall appoint five learned, skilled, and capable veterinary surgeons who shall have been residents of the State of Montana for not less than one year preceding their appointment, not more than two of whom shall be from the same county, and who shall have attended two courses of lectures and are graduates of accredited colleges of veterinary medicine or universities having a veterinary department recognized by the United States Veterinary Medical Association, who shall constitute the Board of Examiners for the purposes of this Act. The veterinarians so appointed shall hold their respective offices for seven years, providing that the term of office of those constituting any present board shall not be affected by the provisions of this Act; and the terms of their successors shall be so arranged as to succeed the present incumbents as their terms expire; and providing, also, that all vacancies occurring shall be likewise filled by appointment by the Governor, by and with the advice and consent of the Senate. Appointments made when the Senate is not in session shall take effect immediately and may be confirmed at the next ensuing session; provided, if the Governor cannot find within the State suitable members, or men that he regards as competent to fill the provisions of this Act, that the now existing Board of Human Medical Examiners, in conjunction with the State Veterinarian, shall examine such applicants as desire to practise veterinary medicine in this State until such time as the Governor can find and appoint a suitable veterinary examining board consisting of veterinarians alone.

The Board of Veterinary Examiners must, on the first Tuesday in January of each year, elect from among their number a president, a secretary, and a treasurer, and must have a seal. Three members of said board shall constitute a quorum. The president and secretary have the power to administer oaths in examination of applicants for certificates and witnesses called before the board in the transaction of business under the provisions of this Act. The Board of Examiners must hold meetings for examinations at the seat of government on the first Tuesdays of January and September of each year, and at such other times and at the same and other places as the board may determine. The board must keep a record of all proceedings thereof; also a register of all applicants for a certificate, with the age of the applicant, time spent in the study of medicine, and the name and location of all the institutions granting to such applicant degrees or certificates of lectures attended in medicine or surgery. Such register shall be *prima facie* evidence of all matters therein kept.

Every person hereafter wishing to practise veterinary medicine or surgery in any of the departments of this State shall apply to said board for a certificate so to do. Every person so applying shall present his diploma to the said Board of Examiners for verification as to its genuineness, and if the diploma is found genuine, and if issued by a veterinary medical school, or the veterinary department of a university legally organized and in good standing, whose teachers are graduates of legally organized schools of veterinary medicine and human medicine, which facts the said Board of Examiners shall determine, and if the person presenting and claiming said diploma be the person to whom the same was originally granted, at a time and place designated by the said board, or at a regular meeting of the said board, said applicant shall submit to an examination in the following branches, to wit: Comparative anatomy, physiology, chemistry, histology, pathology, materia medica, therapeutics, preventive medicine, practice of medicine, surgery, obstetrics, meat- and milk-inspection, hygiene, external form of the horse, zoötechnics, and such other branches as the Board of Medical Examiners may deem advisable, and present evidence of having attended three courses of lectures of at least six months each; but such evidence of having attended three courses of lectures shall not be required of applicants graduating prior to July 1, 1898. Said board shall cause such examinations to be both theoretic and practical and of sufficient thoroughness and severity to test the candidates' fitness to practise veterinary medicine and surgery and dentistry. When desired, such examinations may be conducted in the presence of the dean of any veterinary medical school, or the presidents of any veterinary or human medical society of this State. At the examination such board shall, if the candidate has been found qualified, grant a certificate to such candidate to practise medicine and surgery in the State of Montana, which said certificate can only be granted by the consent of not less than three members of the said board, and which said certificate shall be signed by the president and the secretary of said board and attested by the seal thereof; provided, however, that during the intervening period of the session of the board any person desiring to practise medicine in this State may present his or her diploma to the president of the board, who may issue a certificate good until the next regular meeting of the board; and provided, further, that all veterinary surgeons who hold certificates granted by the now existing Board of Medical Examiners shall be exempt from the provisions of this section.

The board may refuse to grant a certificate for unprofessional, dishonorable, or immoral conduct. Before a certificate can be refused for such a cause the board must serve in writing upon the applicant a copy of any charge or charges against him, and appoint a day for hearing, at which the applicant or any witnesses in his behalf may appear and give testimony in refutation of such charge or charges. In case the board, after hearing such charges, refuse a certificate to the applicant, the decision specifically stating the ground upon which refusal is made must be reduced to writing, and a copy thereof be delivered to the applicant upon demand of the applicant. Upon a like hearing the board may refuse a certificate to anyone who may publicly profess to cure or treat disease, injury, or deformity in such a manner as to deceive the public. The hearing provided for herein must take place within twenty days after the service of a copy of the charges upon the applicant, unless delayed by or further time is granted to the applicant, and the decision of the board must not be later than ten days after the hearing. If the decision is not rendered within the said period of ten days, the applicant is not subject to any penalties for practising without a certificate during the time that elapses before the decision is made. The board, with concurrence of three members thereof, may revoke a certificate for unprofessional, dishonorable, or immoral conduct. Before such revocation can take place a written complaint, specifically stating the charges against the person whose certificate is sought to be revoked, must be delivered to the board and a copy thereof served upon such person twenty days before the time fixed by the board for the hearing of such charges. If, after the hearing, the board revoke the certificate of such person, the ground upon which such revocation is made must be specifically stated by the board in writing and a copy thereof delivered on demand to the person whose certificate is revoked. In all cases of the refusal or revocation of a certificate to practise veterinary medicine by the State Board the person aggrieved thereby may appeal from the decision of the board to the district court of the county in which the refusal or revocation was made. The appeal is taken by the appellant serving notice of appeal upon any officer of the board within ten days after receiving from the board a copy of the decision and filing within the same time with the clerk of the proper district court a verified copy of the decision appealed from, together with a verified copy of such charges furnished by the board and the appellant. The appeal is conducted to a determination in all respects as an appeal from a decision from a board of county commissioners disallowing a claim. The district court must affirm or reverse the decision of the Board of Veterinary Medical Examiners. In case of a reversal a certified copy of the judgment is equivalent to a certificate permitting the applicant to practise veterinary medicine and surgery. An appeal may be taken from the judgment of the district court by either party to the supreme court within sixty days from the entry thereof, in like manner as in civil cases. In case of an appeal from a decision revoking a certificate the appellant may be permitted, in the discretion of the court, to practise during the pendency of the appeal. Every person obtaining a certificate from the board must, within sixty days from the date thereof, have the same recorded in the office of the county clerk where he resides. If he moves from one county to another to practise veterinary medicine or surgery, the certificate must immediately be recorded in the county to which he removes. The county clerk must indorse upon the certificate the date of record, and he is entitled to charge and receive his usual fee for such services, the fee to be paid by the applicant. Until the certificate be recorded, as provided by this section, the physician practising under it is subject to the penalties prescribed in the penal code for practising without a certificate.

This act shall not apply to owners of domestic animals treating their own animals or to owners castrating or spaying their own domestic animals, or to commissioned veterinary surgeons in the United States Army in the discharge of their official duties, or to physicians and surgeons in actual consultation from other States and Territories.

Any person practising veterinary medicine, dentistry, or surgery within this State contrary to the provisions of this act shall, for each violation of the provisions of this act, be guilty of a misdemeanor, and upon conviction shall be fined not more than five hundred dollars nor less than one hundred dollars, or be imprisoned in the county jail for not more than ninety days nor less than thirty days, or by both said fine and imprisonment, as the court may determine.

Any person shall be regarded as practising within the meaning of this act who shall append the letters "D.V.S.," "D.V.M.," "V.S.," "Veterinary Dentist," or any title claiming to be what he is not, or who shall publicly profess to be a veterinary surgeon or physician or dentist, or who shall recommend, prescribe, or direct for the use of any animal, any drug, medicine, appliance, apparatus, or other agency, whether material or not material, for the cure, relief, or palliation of any ailment or disease of whatsoever character, or for the cure or relief of any wound, fracture, or bodily injury or other deformity, after having received, or with the intent of receiving therefor, either directly or indirectly, any bonus, gift, or compensation.

Candidates for examination shall pay in advance to the Secretary of the Board of Medical Examiners a fee of fifteen dollars, which shall defray the entire expenses of the said candidate for examination before the aforesaid Board of Examiners. Anyone failing to pass the required examination shall be entitled to a second examination within six months without fee; and the money so received shall be turned over to the State Treasurer, to be deposited by him in the "Medical Board Fund," as hereinafter provided. Each member of the board shall be entitled to the sum of five dollars per day and actual mileage while in the discharge of his official duties; provided there shall be enough money collected from the candidates examined to pay such expenses and mileage. The entire expenses of said Veterinary Medical Examining Board shall only be paid out of funds paid into the State treasury by candidates examined.

The board must report annually on the first Monday in January to the Governor, which report must show all the transactions of the board, giving the number of the applications received and from whom received, the number of certificates granted and revoked, and the names of those receiving certificates, and those rejected, giving the reasons therefor, the amount of money received, the expense of the office and mileage paid, and by whom received, and the amount of money remaining in the said fund.

The veterinarians now practising within this State who have been practising within the limits of the State for a period of three years or over, who are graduates in good standing of veterinary colleges or veterinary departments of recognized universities, shall only be compelled to pass a practical examination in the branches herein mentioned.

This act shall be in force and effect on and after its passage and approval by the Governor.

### A Bill for An Act Providing for the Appointment of a Meat-inspector in Cities having Ten Thousand Inhabitants or over within the State of Montana, and Prescribing the Duties and Powers of such Inspector, and to Regulate the Sale of Stock, Milk, and Meat for the Purposes of Food, and Prescribing Penalties for the Violation of the Provisions of this Act.

*Be it Enacted by the Legislative Assembly of the State of Montana:*

Section 1. The office of meat-inspector is hereby created in cities having a population of ten thousand inhabitants or over within the State of Montana; and immediately after taking effect of this act such cities shall appoint a meat-inspector or inspectors, whose compensation shall be borne by said cities, and shall be such as shall secure the services of some competent and qualified person, who shall take an oath of office to faithfully perform the duties of his office and to execute an official bond to the said city in the sum of five thousand dollars. No person shall be appointed a meat-inspector unless he is a graduate in good standing from some regular and reputable veterinary or medical college, admitted to practise in Montana, and before that appointment he shall be required to exhibit his diploma as such graduate.

SEC. 2. It shall be the duty of the city council of all cities having a population required by this act to designate some place or places in or adjacent to the city where the cattle, sheep, or swine intended for meat-slaughter, sale, and consumption for food in said city shall be brought for inspection on the hoof, where the meat of any said animals may be brought for inspection, which inspection shall be made without any unreasonable delay; and no fee or charge shall be made against or demanded of the owner or person who shall present any such animal or animals or meat for such inspection, but the same shall be inspected free of any expense whatever for or on account of the services of such inspector. And it is hereby made the duty of such inspectors to keep a correct record in a suitable and substantial book provided for that purpose, in which he shall record the names, places of residence, post-office address of the owner or owners of all animals presented for inspection, together with the brands and marks and a full description thereof, and the owner or owners of all meat which shall be presented for inspection, and also the names, places of residence, and post-office address of the persons from whom the same were purchased by the person or persons who may present the same for inspection.

SUBDIVISION 2. The rules, regulations, and methods of inspection adopted by the Bureau of Animal Industry, conducted by the United States Government, shall be taken as the standard of meat-inspection, and shall be followed as closely as may be practical by the meat-inspectors appointed by said cities.

SEC. 3. First. All meat intended to be offered for sale and consumption for food, and all cattle, sheep, and swine intended for immediate slaughter, sale, and consumption for food in cities having the population required by this act, shall be submitted to the meat-inspector on the day immediately preceding the day on which the same shall be slaughtered, and shall be taken to such place or places as shall be provided and designated under the provisions of Section 2 of this act, for such inspection.

Second. The carcasses of all animals so inspected on the hoof and slaughtered shall be inspected before the same shall be exposed or offered for sale, and such carcasses, and also all meats of any of the animals mentioned in this act, where the animals shall not have been presented for inspection on hoof before being slaughtered, shall be inspected before being offered or exposed for sale, and such carcass or carcasses or meat as shall be found upon such inspection and examination to be wholesome and fit for food shall be marked by the inspector with a tag similar in form and character to that used by the Government of the United States in the Bureau of Animal Industry, which tag shall be adopted and designated by the city council of said cities as the city stamp or certificate for the designation of wholesome and healthy meats;

Provided: That nothing herein contained shall be so construed as to prevent any person from slaughtering without inspection any healthy animal, the meat of which is intended for his own use or that of his family and household.

Sec. 4. It shall be the duty of the inspector to make inspection of the meats, carcasses, and animals mentioned in this act which may be presented for inspection at the place or places designated by the city council and keep the record aforesaid in the manner and form herein provided, which inspections shall be made by him as soon as possible and without unreasonable or unnecessary delay; and he shall attach to all meats so inspected and examined and found healthy and wholesome and fit for food a tag indicating that fact.

Sec. 5. The meat-inspectors appointed by said cities shall have the right to condemn any meat, carcass or carcasses, or parts thereof, of all cattle, sheep, or swine, or any other articles of food which they may find after examination to be unfit for food, and shall order the owner to destroy the same in such manner as may be acceptable to said inspector.

Sec. 6. It shall be unlawful to sell or offer for sale, buy or offer to buy, within the limits of said city, for the purpose of food, any calf, or the carcass or part of carcass of any calf, under the age of thirty days, or any cow, or the carcass or part of carcass of any cow, that have calves inside with hair on; and it shall be the duty of such inspector to condemn such cows and calves, and the carcasses of such cows and calves, at the time the same are inspected, and cause the same to be destroyed as aforesaid.

Sec. 7. Any person or persons, company or corporations, who shall sell or offer for sale, buy or offer to buy within the limits of said city, any carcass or carcasses or portion thereof, of any cattle, sheep, or swine, which have not been inspected or tagged as herein required or accepted as herein stated, or shall violate any of the provisions of this act, shall be guilty of a misdemeanor, and upon conviction thereof be punished by a fine not exceeding two hundred ($200) dollars.

Sec. 8. Provided: That nothing in this act or any paragraph thereof shall be construed to interfere with the offering for sale of any meats bearing a stamp or tag indicating that the same has been inspected by the United States Government or some county municipality where the proper sanitary meat-inspection, as required by this act, has been complied with.

Sec. 9. Said meat-inspector shall also be ex-officio inspector of milk, and as such it shall be the duty of said meat- and milk-inspector to inspect each dairy supplying milk to such city not less than once in each month of the calendar year. Each dairy shall have a certificate of health from said inspector for each cow from which the public is supplied with milk.

Sec. 10. It shall be unlawful for any dairyman to feed any unwholesome food of whatsoever character, and for each offence shall be fined within the penalty provided by this act. Each dairyman supplying milk to the public must have for each cow a certificate of having had the tuberculin-test applied by said inspector, stating that said cow is free from tuberculosis or consumption or any infectious or contagious disease whatsoever. Any dairyman having in his own family a case of diphtheria or scarlet fever *or any infectious* disease that may or might contaminate his milk is prohibited by this act from selling milk to the public for such a period as the disease exists in his or her family, and for twenty (20) days after said disease has subsided, and the inspector has given certificates that the premises have been thoroughly disinfected.

SEC. 11. All milk supplied by the public dairies shall contain not less than three per cent. (3 per cent.) of butter fats, and shall come up to a normal specific gravity test for milk.

SEC. 12. When, in the belief of the inspector, proper cleanliness of the utensils, buckets, pans, and other articles used about accumulating and in the marketing of milk are not up to the standard, it shall be the inspector's duty to destroy such milk and prohibit the said dealers from selling milk until such times as proper cleanliness and precaution are used in its handling.

SEC. 13. Dairymen in such dairies as supply milk to said cities must keep their barns or stables free from filth or dirt accumulating within or about their stables, that are likely to generate dirt, or filth, or disease-producing germs that can be carried within the milk.

SEC. 14. It shall be the duty of said inspector to stop at any time he may deem fit a dairy wagon or any vehicle containing milk that is intended for public consumption, and there and then take cognizance of any irregularity of whatsoever character in the milk or its methods of handling or distribution; ascertain if it is not up to the regular standard or that recognized standard to which milk should come, and if he finds said milk deficient in any of its nutritive qualities or sufficiently uncleanly to jeopardize the health of the people by whom it is consumed, he shall there and then condemn such milk, and such dairyman or milkman or other person whose product has been condemned shall be prohibited from selling any milk until he shall have received his certificate from said inspector permitting him to do so.

AMENDMENT TO SECTION 14. Provided: That such inspector shall, if requested by such dairyman, take from the same can of milk from which he shall have taken any quantity of milk, for the purpose of testing the same, at least one pint of milk and place the same in a bottle and seal and mark the same in such manner as to identify the same, and deliver the same to such dairyman, who may have the same analyzed and tested by any person competent to test and analyze the same, in order that such dairyman may ascertain the correctness of the inspector's analysis of such milk.

SEC. 15. Any citizen of the State of Montana to whose knowledge or observation comes the fact that any dairyman is supplying milk from diseased cattle or cattle fed on manure and refuse of a stable or other improper feed, it shall be his duty to notify said inspector, who shall at once visit said dairy, and if he find complaint to be true, it shall be his duty to at once prohibit the further selling of the product of said dairy and at once file an information against the said dealer in the nearest court.

SEC. 16. This act shall apply to all products of the dairy where sold in this State or any municipality to which the district covered by the inspector belongs.

SEC. 17. It shall be unlawful to adulterate milk in any manner whatsoever by drugs, coloring-matter, or any extraneous substance which, in the judgment of the inspector, would be detrimental to public health. Any method of handling milk intended for the public likely to produce unwholesome changes in the milk or disease to the consumers shall be prohibited from exposure for sale, and a violation of this section shall be punished as is above mentioned within the meaning of this act. The use of any drug or any unnatural method whatsoever of preservation or changing of milk shall be punished within the provisions of this act.

SEC. 18. Said meat- and milk-inspector shall perform such other duties in inspecting articles of food as the city council may require; provided that such additional duties shall not interfere with the prompt and proper discharge of the duties of meat- and milk-inspector as required and specified in this act.

SEC. 19. Said meat- and milk-inspectors shall have the same power as is now conferred on sheriffs, constables, and other police officers to arrest any person or persons whom said meat- and milk-inspectors may have reason to believe guilty of violating any of the provisions of this act.

SEC. 20. Any person or persons who shall forge, counterfeit, or knowingly or wrongfully utter, deface, or destroy, or lose any of the marks, stamps, or other devices which may be adopted by said cities as herein required for the purpose of designating wholesome or healthy meats or milk, or sell or attempt to sell any meats or milk which may have been condemned by the said meat- and milk-inspectors, shall be deemed guilty of a misdemeanor within the meaning of this act, and upon conviction thereof shall be punished by a fine not exceeding three hundred dollars, or by imprisonment not exceeding six months, or by both such fine and imprisonment, in the discretion of the court.

SEC. 21. Any city in the State of Montana having a population of less than ten thousand inhabitants shall have the option of adopting the sanitary provisions of this act.

SEC. 22. This act shall take effect and be in force from and after the first day of July, 1897.

SEC. 23. All acts and parts of acts in conflict herewith are hereby repealed.

